# Quality expectations and tolerance limits of trial master files (TMF) – Developing a risk-based approach for quality assessments of TMFs

**DOI:** 10.3205/000227

**Published:** 2015-12-10

**Authors:** Arthur Hecht, Barbara Busch-Heidger, Heiner Gertzen, Heike Pfister, Birgit Ruhfus, Per-Holger Sanden, Gabriele B. Schmidt

**Affiliations:** 1 Global Quality Medicine, Boehringer Ingelheim Pharma GmbH & Co. KG, Biberach, Germany; 2 Quality Medicine Germany, Boehringer Ingelheim Pharma GmbH & Co. KG, Biberach, Germany; 3 R&D Clinical & Medical Quality Operations, Sanofi, Chilly-Mazarin, France; 4 Clinical Project Management Specialty Medicine, Bayer Pharma AG, Berlin, Germany; 5 Global Inspection Management, Merck Serono, Darmstadt, Germany; 6 Global Clinical Trial Operations, MSD SHARP & DOHME GMBH, München, Germany

**Keywords:** trial master file, quality risk management, clinical trial

## Abstract

This article addresses the question of when a trial master file (TMF) can be considered sufficiently accurate and complete: What attributes does the TMF need to have so that a clinical trial can be adequately reconstructed from documented data and procedures?

Clinical trial sponsors face significant challenges in assembling the TMF, especially when dealing with large, international, multicenter studies; despite all newly introduced archiving techniques it is becoming more and more difficult to ensure that the TMF is complete. This is directly reflected in the number of inspection findings reported and published by the EMA in 2014.

Based on quality risk management principles in clinical trials the authors defined the quality expectations for the different document types in a TMF and furthermore defined tolerance limits for missing documents. This publication provides guidance on what type of documents and processes are most important, and in consequence, indicates on which documents and processes trial team staff should focus in order to achieve a high-quality TMF.

The members of this working group belong to the CQAG Group (Clinical Quality Assurance Germany) and are QA (quality assurance) experts (auditors or compliance functions) with long-term experience in the practical handling of TMFs.

## Zusammenfassung

Der Artikel setzt sich mit der Frage auseinander, wann ein Trial Master File (TMF) als ausreichend genau und vollständig angesehen werden kann. Wie muss ein TMF beschaffen sein, um anhand der dokumentierten Daten und Prozesse eine klinische Prüfung hinreichend rekonstruieren zu können?

Die Zusammenstellung eines TMF ist für Sponsoren von klinischen Prüfungen eine erhebliche Herausforderung. Dies trifft insbesondere bei großen internationalen multizentrischen Studien zu. Trotz modernster Archivierungsmethoden wird es immer schwieriger sicherzustellen, dass ein TMF vollständig ist. Das zeigt sich ganz direkt an der Zahl der Inspektionsbeobachtungen, welche die EMA 2014 publiziert hat.

Basierend auf den Prinzipien eines risikobasierten Qualitätsmanagements für klinischen Prüfungen haben die Autoren für die unterschiedlichen Dokumenttypen eines TMF die Qualitätsanforderungen definiert und Toleranzgrenzen für fehlende Dokumente festgelegt. Der Artikel gibt Hilfestellung für die Entscheidung, welche Dokumenttypen und Prozesse besonders wichtig sind und auf welche Dokumente und Prozesse in der Konsequenz die Studienteams besonderes Augenmerk legen müssen, um einen qualitativ hochwertigen TMF zusammenzustellen.

Die Arbeitsgruppe besteht aus Mitgliedern der CQAG (Clinical Quality Assurance Germany) und QA-Experten (Auditoren oder Compliance Funktionen) mit langjähriger Erfahrung in der praktischen Handhabung von TMFs.

## Introduction

In February 2013, the European Medicines Agency (EMA) published the “Reflection paper on GCP compliance in relation to trial master files (paper and/or electronic) for management, audit and inspection of clinical trials”. This paper was the EMA’s response to a question from the EMA Inspection Working Group on how to handle the drastic increase in minor and major inspection findings in the trial master file (TMF) from 2011 to 2012, and in particular how to address TMF quality issues in the future [1], [2], [3], [4]. 

The essential documents in the ICH GCP (International Conference on Harmonization of technical requirements for registration of pharmaceuticals for human use – Good Clinical Practice), published in 1996 [5], is the minimum list of documentation but is not a comprehensive content list for the TMF [2]. From 1996 to the present, the environment of clinical trials has changed drastically, requiring additional documentation in the TMF. All documents associated with a clinical trial that are needed to reconstruct the course and conduct of the trial are relevant for inclusion in the TMF [2]. Therefore the core documents as listed in the Drug Information Association (DIA) TMF Reference model Version 2 (2012) [6] were considered more germane and are included in the risk assessment that we performed (see below).

Inspectors from the EMA have frequently identified problems with the TMF, including that sponsors often fail to provide a comprehensive TMF. The EMA stated in its “Reflection paper on GCP compliance” that TMFs should be complete and accurate [2]. This raises the question: When can a TMF be considered accurate and complete? At present, no quality characteristics have been defined to ensure a TMF is up to standard. The EMA “Reflection paper on risk-based quality management in clinical trials” requested that tolerance limits should be established [7]. However, at present no detailed regulatory guidance is available with regard to tolerance limits, nor are we aware of any publication proposing acceptable tolerance limits for the completeness of a TMF. 

The main objective of this cross-company working party was to establish explicit expectations as to when a TMF can be accepted as sufficiently accurate and complete. Risk management methods were employed to define quality expectations and tolerance limits.

## Approach for developing quality and tolerance limits for TMFs

The impact on “Safety, rights and wellbeing of patients” as well as “data integrity” was used as the basis for the risk assessments. This is in accordance with the EMA’s view that these are the ultimate principles in GCP and that they should guide the assessments of quality in clinical trials [7]. 

As a first step, a risk assessment was performed for all 148 required types of core documents included in the DIA TMF Reference model Version 2 (2012) [6] and provided as data publication at Dryad [8]. For each document type the impact with regard to “Safety, rights and wellbeing of patients” as well as “data integrity” was determined based on the assumption that a missing document would indicate that the underlying process was not performed (worst case scenario). 

The team members assessed the impact of the missing process on patient rights and safety and trial data integrity, using a 10-point scale. The impact was rated as “critical” (score between 8 and 10) if the missing process would have a *direct* effect on patient rights and safety or trial data integrity. “Major” (score between 5 and 7) was used if it would have *possible* effects on patient rights and safety or trial data integrity. A score between 2 and 4 was chosen for a “minor” impact that would have *no expected* effects on patient rights and safety or trial data integrity. Finally a score of 1 was applied if the missing process would not have any effect on patient rights and safety or trial data integrity but only impact the documentation of the clinical trial. Examples of each type of document are provided in Table 1 (Tab. 1). The full list of all assessed document types is provided as data publication at the Dryad repository (see [8]).

**Figure 1:**
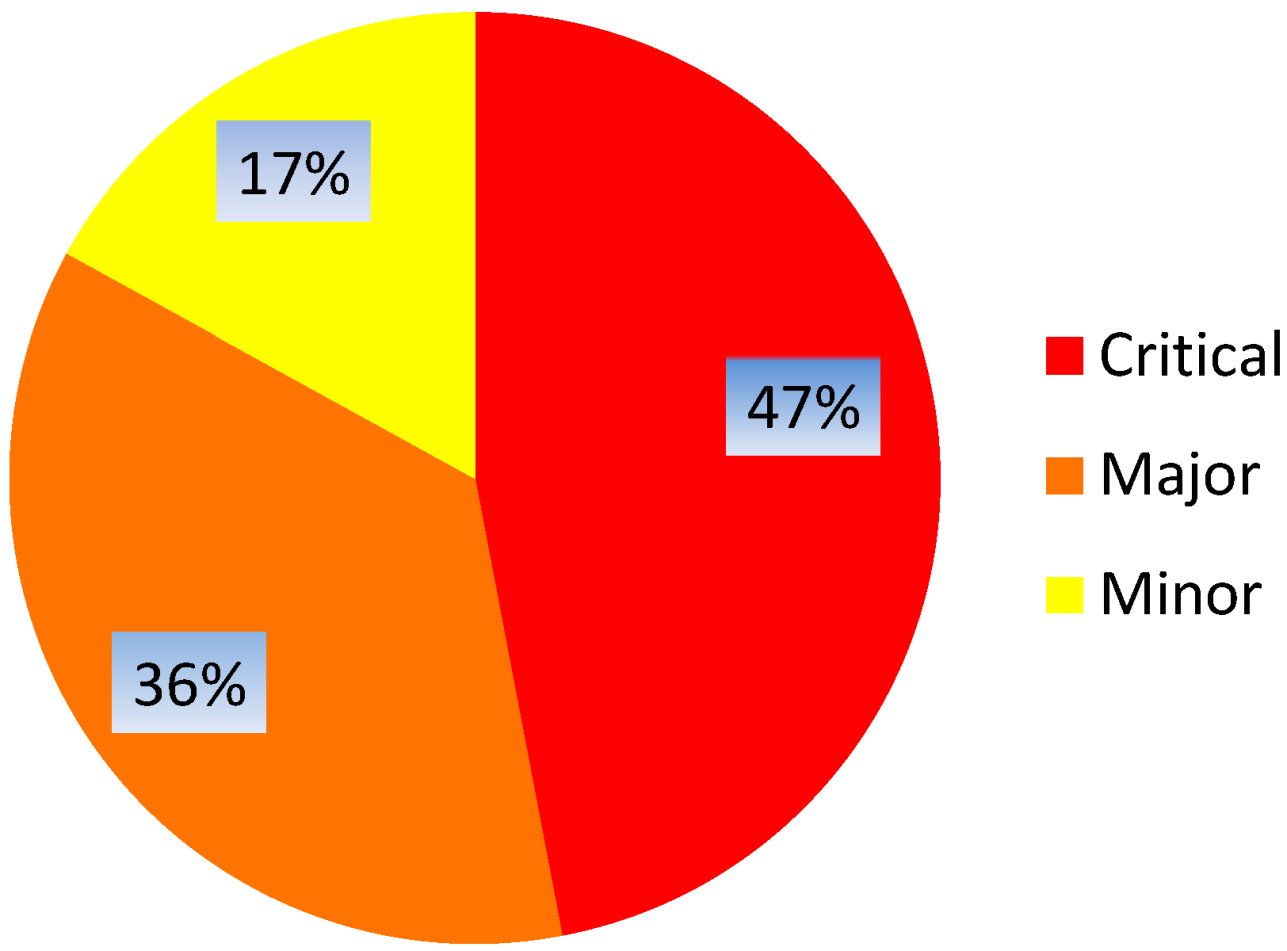
Distribution of the impact of missing document types/processes on patient rights and safety and/or trial data integrity

As stated above, 148 types of documents were included in the risk analysis. Sixty-nine types of documents (47%) were categorized as critical, 54 types of documents (36%) were categorized as of major importance, and 26 types of documents (18%) were categorized as of minor importance, as their absence was not expected to have impact on patient rights, safety or data integrity (Figure 1 (Fig. 1)).

In a second step a risk assessment was performed to determine how much effort would be required to replace or substitute a missing document in the TMF, assuming that the associated process was performed for the clinical trial (that is, that the document had been generated during the trial but was not available in the TMF).

For all core documents assessed in step 1 the level of effort required for tracing or replacing the document was assessed. If the original was available the document type received a risk score of 1; if a copy could be filed in the TMF, the missing document received a score of 2. When the document was missing, but the process could be proved to have taken place using other documents, the effort of this verification was assigned a score of 3 to 6.

### Overall risk assessment

The overall risk assessment combined the results of step 1 (the impact of a process that was not performed) with step 2 (the effort to replace or substitute a document). The overall risk assessment was calculated using the following formula:

Risk Priority Number (RPN) = (impact of missing process)² × effort to replace the document

**Figure 2:**
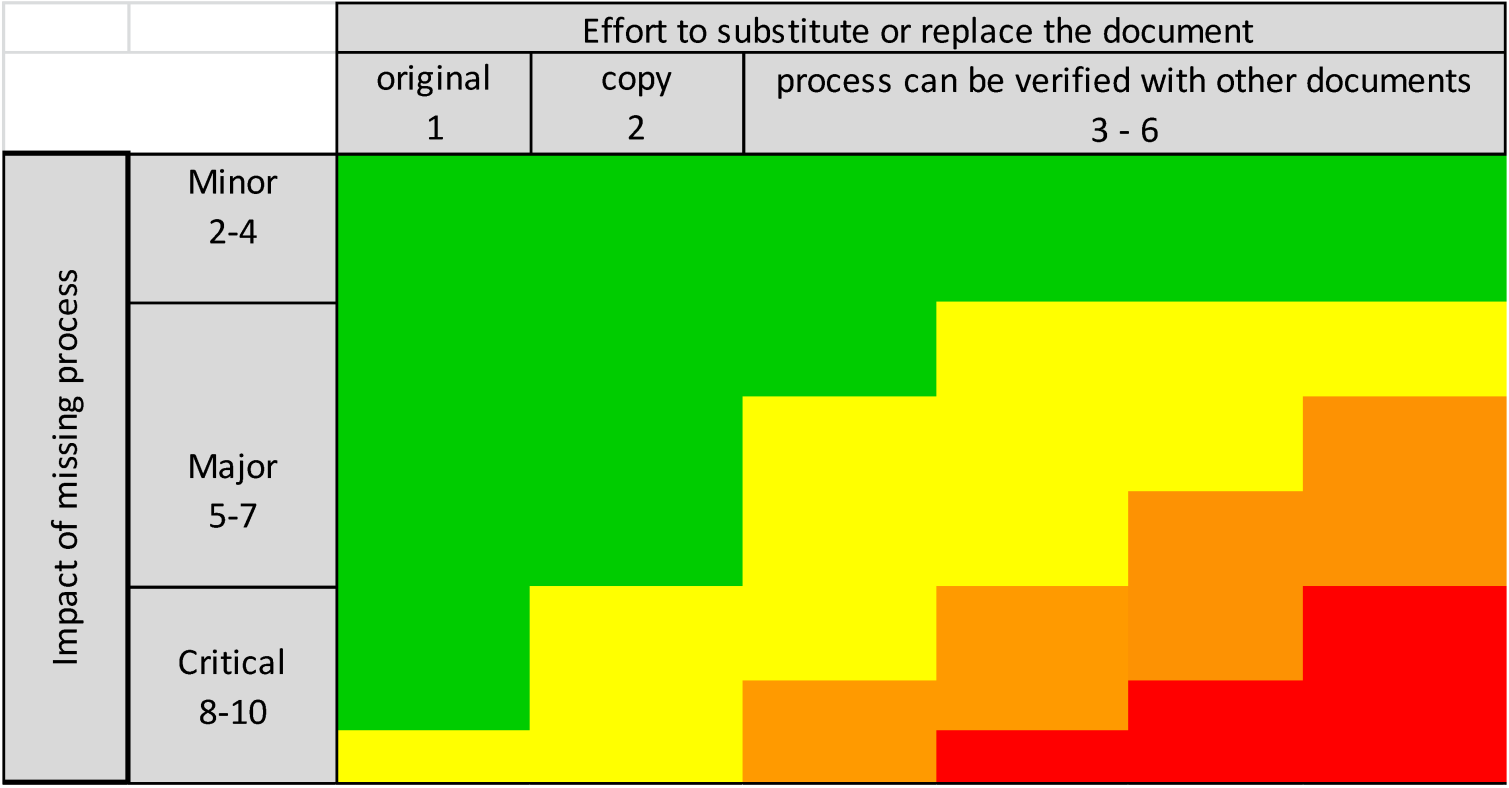
Overall risk-rating matrix

Because the importance of patient rights and safety was considered much more important than the effort required to substitute or replace a document, the score for the impact of the missing process was squared in the formula above. The resulting risk priority number (RPN) was plotted on a risk-ranking matrix (Figure 2 (Fig. 2)) in order to assess the overall risk category. The numerical values of the risk categories are displayed in Table 2 (Tab. 2).

**Figure 3:**
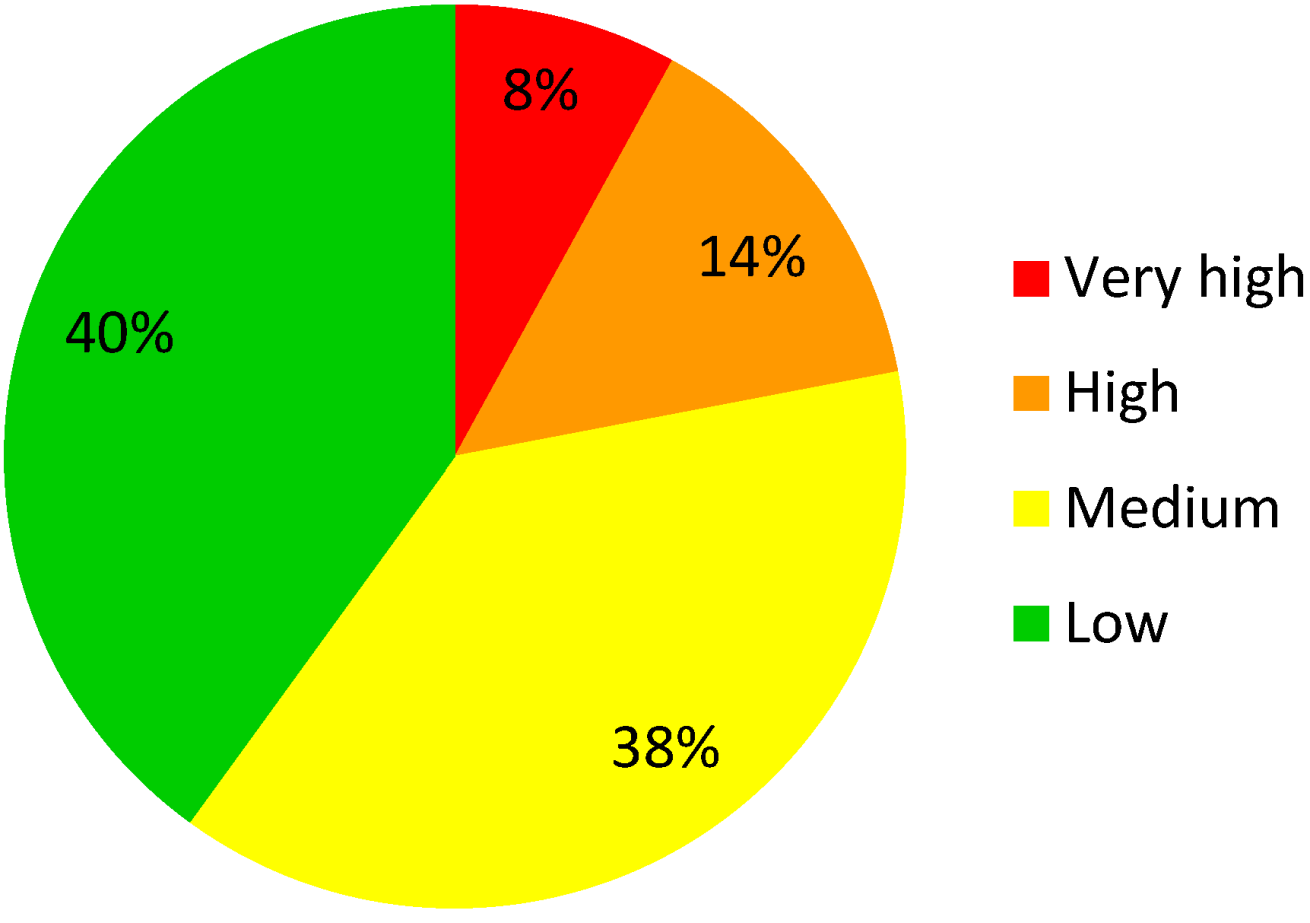
Distribution of the document types according to the overall risk categories

Categorization of all 148 required types of core documents in the DIA TMF Reference model indicated that only 8% of all document types in a very high risk category, whereas 14% are in a high risk category, 38% of the document types are in a medium risk category, and 40% of all document types are in a low risk category (Figure 3 (Fig. 3)). 

## Setting of tolerance limits for missing documents in the TMF

Based on the first risk assessment step in which the impact of missing documents/processes on patient rights and safety and/or trial data integrity were assessed, the team specified tolerance limits. The tolerance limit indicated what level of completeness is necessary to ensure acceptable quality of a TMF. The tolerance limit for “stand alone documents” (for instance, the clinical trial protocol) was 0%. Table 3 (Tab. 3) shows the defined tolerance levels.

To illustrate the effect of the defined quality expectations, the team estimated the number of documents for a trial with 100 sites and calculated the acceptable number of missing documents according to Table 3 (Tab. 3). For instance, assuming each site was provided investigational material three times during the trial, this would amount to 300 documents. The quality expectation of <1% would still be fulfilled if 2 documents were missing (Table 4 (Tab. 4)). 

The full list of all assessed document types is provided as data publication at the Dryad repository (see [8]).

## Discussion

The TMF should adequately document trial processes and thereby ensure that patients’ rights are respected, that their safety is assured, and that the trial data are reliable. Not all documents in the TMF are of equal value in documenting these outcomes. The absence of some documents (and their associated processes) may have a critical impact on these outcomes, whereas others may have almost no impact at all. Therefore, when considering how to assure an adequate-quality TMF, it is important to assess the importance of the individual documents rather than to simply consider the overall number of documents filed. Therefore, tolerance limits for missing documents cannot be specified uniformly and should be assessed based on the impact on patient rights and safety and trial integrity. Our approach determines the importance of a missing document, and in addition (if the trial process has been performed, but the document is absent), indicates the amount of effort required to replace it. We have generated a list of document types of very high and high importance which should be focused on in order to assure an adequate high-quality TMF, while on the other hand identifying lower risk areas which require less emphasis and attention during quality control steps without endangering the integrity of the entire TMF. This list could be of major assistance to anyone working with TMFs, e.g. helping to ensure adequate and continuous TMF maintenance or to prioritize efforts in an inspection preparation in case of short timelines and limited resources.

The team also rated other deficiencies (e.g. poor scanning quality) observed in QC checks. Respective quality expectations, tolerance limits and respective QC procedures are not included here but will be published in a separate article as it requires a comprehensive treatment.

## Data

Data for this article are available from the Dryad Repository: http://dx.doi.org/10.5061/dryad.t2f61 [8].

## Notes

### Competing interests

The authors declare that they have no competing interests.

### Acknowledgements

We acknowledge Dr. Jill Holbrook who provided medical writing services on behalf of BI Pharma GmbH & Co. KG.

## Figures and Tables

**Table 1:**
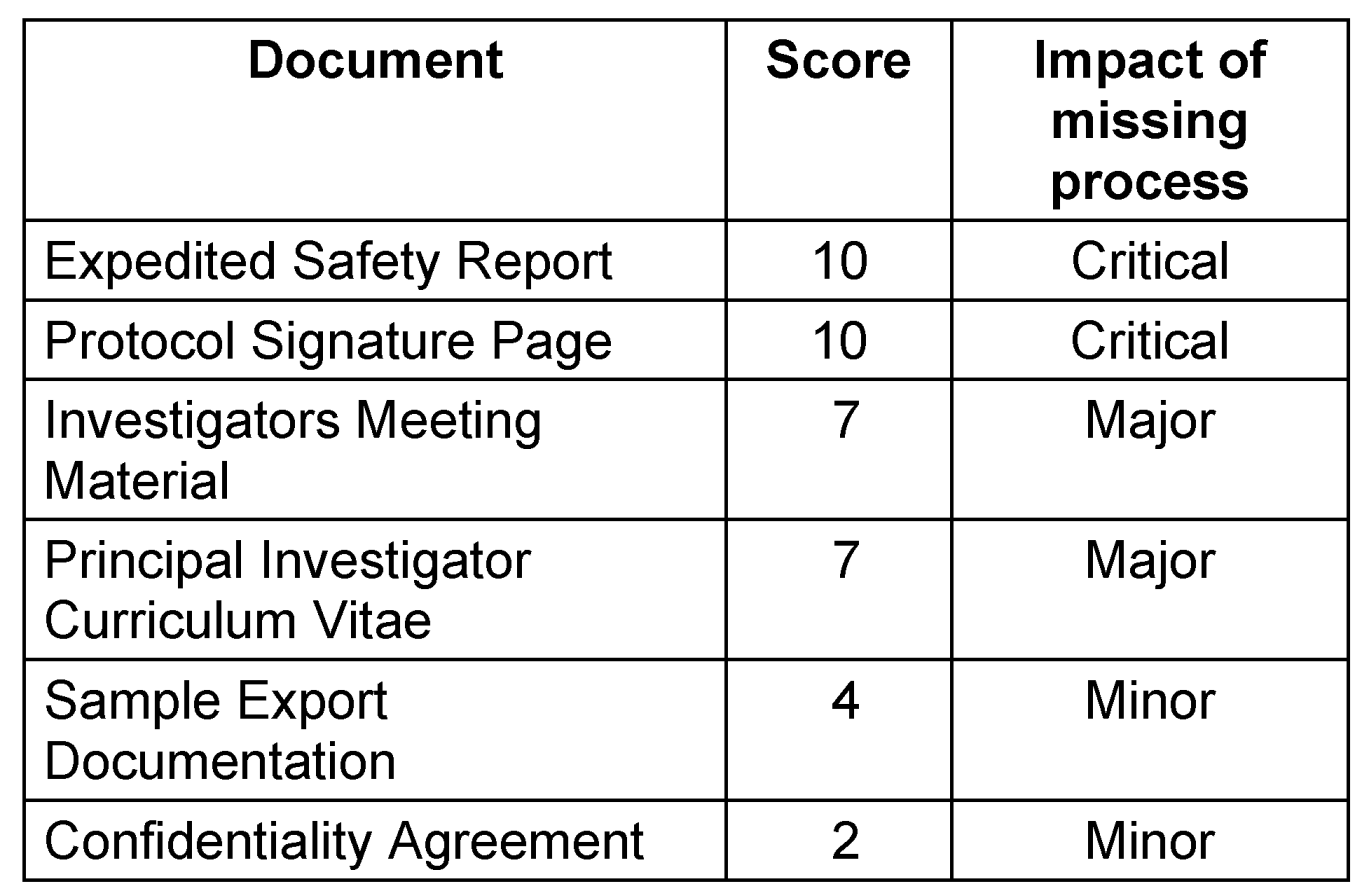
Examples of the impact of missing documents/processes on patient rights and safety and/or trial data integrity

**Table 2:**
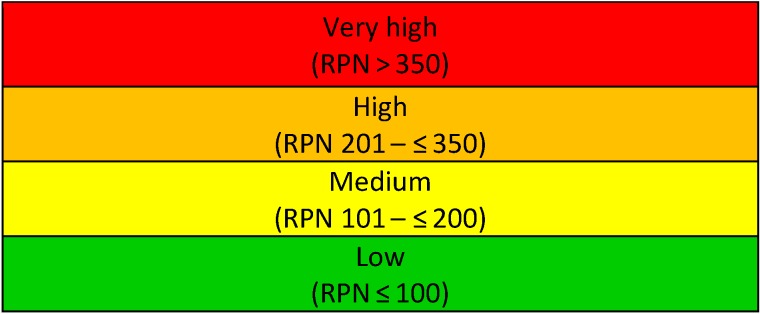
Thresholds for the defined overall risk categories

**Table 3:**
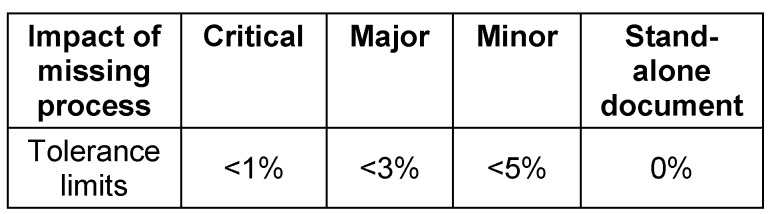
Assigned tolerance limits for documents in the TMF based on the impact of the missing process on patient rights and safety and trial data integrity

**Table 4:**
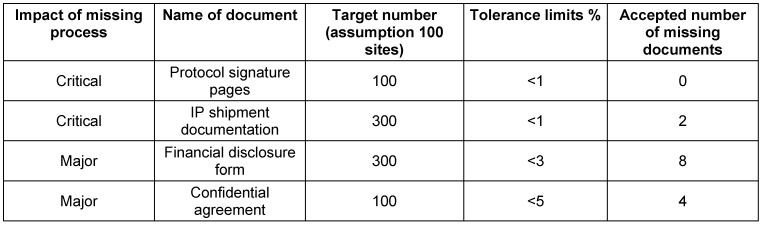
Examples of acceptable number of missing documents for a trial with 100 sites
